# Stability of Maleimide-PEG and Mono-Sulfone-PEG Conjugation to a Novel Engineered Cysteine in the Human Hemoglobin Alpha Subunit

**DOI:** 10.3389/fchem.2021.707797

**Published:** 2021-07-26

**Authors:** Chris E. Cooper, Matthew Bird, XiaoBo Sheng, Ji-Won Choi, Gary G.A. Silkstone, Michelle Simons, Natalie Syrett, Riccardo Piano, Luca Ronda, Stefano Bettati, Gianluca Paredi, Andrea Mozzarelli, Brandon J. Reeder

**Affiliations:** ^1^ School of Life Sciences, University of Essex, Colchester, United Kingdom; ^2^ Abzena Ltd, Cambridge, United Kingdom; ^3^ Department of Medicine and Surgery, University of Parma, Parma, Italy; ^4^ Institute of Biophysics, National Research Council, Pisa, Italy; ^5^ SITEIA.Parma, University of Parma, Parma, Italy; ^6^ Department of Food and Drug, University of Parma, Parma, Italy

**Keywords:** hemoglobin, hemoglobin based oxygen carrier, oxygen therapeutic, maleimide-PEG, mono-sulfone-PEG, cysteine, PEGylation

## Abstract

In order to use a Hemoglobin Based Oxygen Carrier as an oxygen therapeutic or blood substitute, it is necessary to increase the size of the hemoglobin molecule to prevent rapid renal clearance. A common method uses maleimide PEGylation of sulfhydryls created by the reaction of 2-iminothiolane at surface lysines. However, this creates highly heterogenous mixtures of molecules. We recently engineered a hemoglobin with a single novel, reactive cysteine residue on the surface of the alpha subunit creating a single PEGylation site (βCys93Ala/αAla19Cys). This enabled homogenous PEGylation by maleimide-PEG with >80% efficiency and no discernible effect on protein function. However, maleimide-PEG adducts are subject to deconjugation via retro-Michael reactions and cross-conjugation to endogenous thiol species *in vivo*. We therefore compared our maleimide-PEG adduct with one created using a mono-sulfone-PEG less susceptible to deconjugation. Mono-sulfone-PEG underwent reaction at αAla19Cys hemoglobin with > 80% efficiency, although some side reactions were observed at higher PEG:hemoglobin ratios; the adduct bound oxygen with similar affinity and cooperativity as wild type hemoglobin. When directly compared to maleimide-PEG, the mono-sulfone-PEG adduct was significantly more stable when incubated at 37°C for seven days in the presence of 1 mM reduced glutathione. Hemoglobin treated with mono-sulfone-PEG retained > 90% of its conjugation, whereas for maleimide-PEG < 70% of the maleimide-PEG conjugate remained intact. Although maleimide-PEGylation is certainly stable enough for acute therapeutic use as an oxygen therapeutic, for pharmaceuticals intended for longer vascular retention (weeks-months), reagents such as mono-sulfone-PEG may be more appropriate.

## Introduction

The red blood cell (erythrocyte) provides both volume support and oxygen transport. Blood loss during trauma and/or pathology can therefore have serious consequences for the mammalian vasculature, dropping blood pressure and compromising oxygen delivery to tissue. Hemoglobin (Hb) is an oxygen transport protein, present in high concentrations in the erythrocyte (about 5 mM) and responsible for its oxygen transport function. When Hb levels drop below a critical level, the “transfusion trigger” generally between 7 and 9 g/dL, blood transfusions are recommended to restore these capacities (Yao et al., 2020). However, even in developed countries with sophisticated blood banks, systems can become insufficient to meet the demand in crisis situations (Gniadek et al., 2020); a condition that is even more critical in developing countries where it is more challenging to provide a safe supply of red blood cells (Aneke and Okocha, 2017). Therefore, there has long been a desire to create artificial “blood substitutes” that could complement blood transfusion services by providing a long lasting, stable, pathogen free alternative to blood in critical care situations (Winslow, 2006). More recently there has been interest in the development of “oxygen therapeutics”, Hb solutions designed not to replace blood, but to act in synergy with erythrocytes to deliver oxygen to tissues difficult for red blood cells Hb to access (Bettati and Mozzarelli, 2011). Such oxygen therapeutics have been suggested to play a critical role in new therapeutic interventions in, for example, stroke, trauma and sickle cell disease (Kawaguchi, 2017).

By far the most common starting material for a potential blood substitute or oxygen therapeutic is the Hb molecule, either purified from outdated human blood, animal blood or produced using recombinant technology. However, injecting extracellular Hb alone is ineffective. Although the 64 kDa tetramer is large enough to remain in the vasculature, outside the erythrocyte membrane it readily breaks down into dimers which are rapidly cleared by the kidney (Bunn et al., 1969), causing oxidative damage in the process (Feola et al., 1990). Therefore converting Hb into a Hemoglobin-Based Oxygen carrier (HBOC) requires modifications such as chemical or genetic cross-linking, conjugation or encapsulation (Bettati and Mozzarelli, 2011).

The most common conjugation approach used to formulate a HBOC has been PEGylation with a number of PEGylated Hb products being tested *in vivo*. PEGylation of human Hb was used in previous clinical trials by Sangart [MP4, Hemospan (Vandegriff and Winslow, 2009)], APEX Pharmaceuticals [PHP (Vincent et al., 2015)] and Baxter Healthcare [rHb2.0 (Resta et al., 2002)]. Prolong Pharmaceuticals are currently trialling PEGylated bovine Hb product Sanguinate® (Abuchowski, 2017) for the treatment of sickle cell disease. Many of these products used maleimide-PEG conjugation to create the protein-PEG product. However, due to the scarcity of reactive cysteine residues in Hb, reagents such as 2-iminothiolane were used to introduce thiol reactivity at terminal amino residues and surface lysines in Hemospan (Vandegriff et al., 2003), Euro-PEG-Hb (Portoro et al., 2008) and Sanguinate® (Nho et al., 1992). However, this final product is heterogeneous, due to differences in the efficiency of reaction at different protein sites both in creating free thiol residues and/or the subsequent PEGylation at those residues.

The native human Hb α2/β2 tetramer contains six cysteine residues, each α subunit having one at αCys104 and each β subunit having two at βCys93 and βCys112. However, by far the most reactive to chemical modifications is βCys93 (Guidotti and Konigsberg, 1964; Rosemeyer and Huehns, 1967). We recently took advantage of the opportunities afforded by the use of recombinant Hb as an HBOC starting material, by replacing βCys93 with alanine and introducing a novel reactive cysteine residue in the surface of the α subunit at residue 19. The resultant protein (βCys93Ala/αAla19Cys) - hereafter named A12 - enabled efficient homogenous PEGylation at a single protein site (αAla19Cys) with no deleterious effect on protein function (Cooper et al., 2020).

**FIGURE 1:**
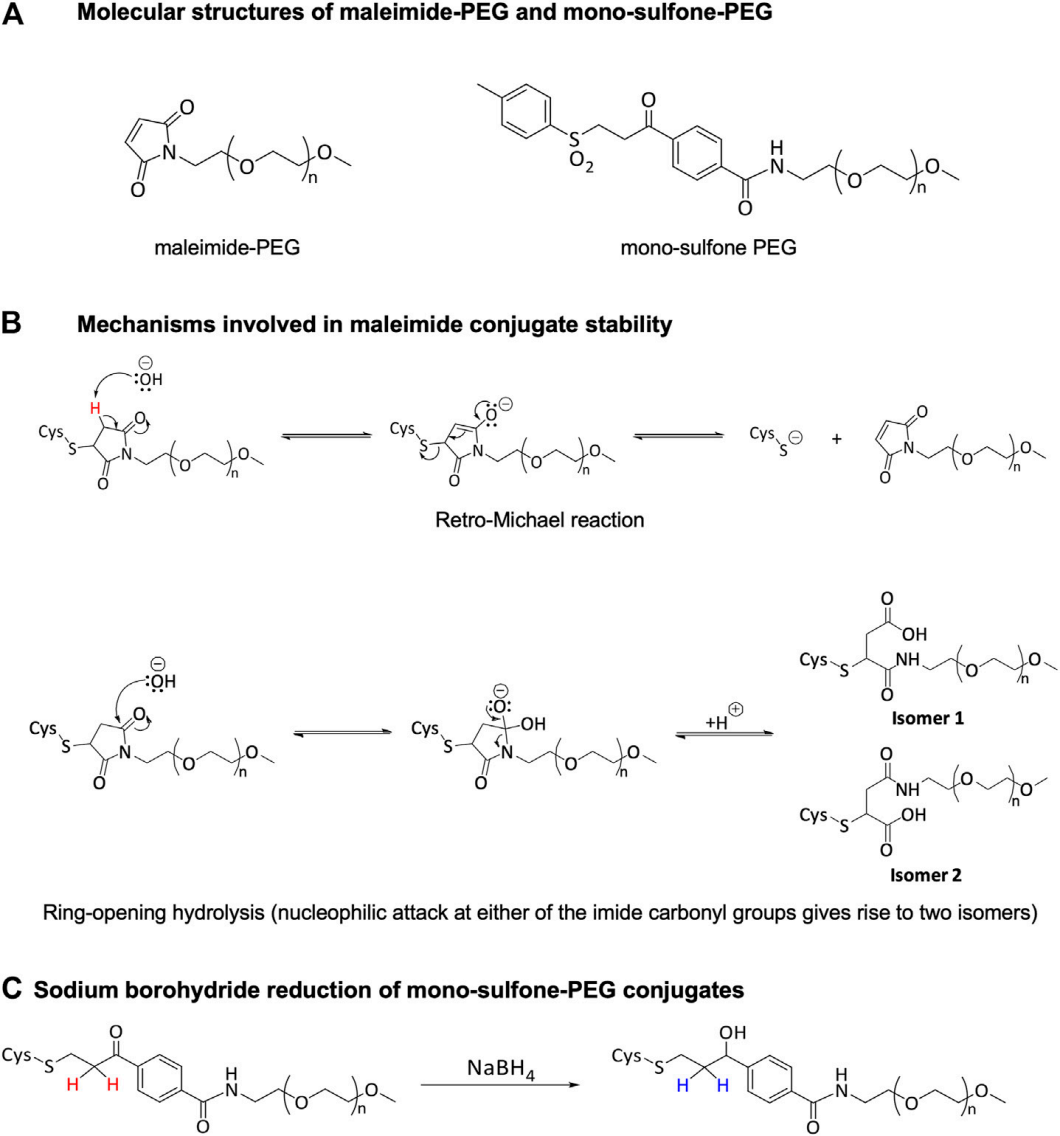
Structure and reactivity of PEG conjugation reagents.

In our original paper, we used maleimide-PEG as the conjugating reagent via Michael addition reactions to the free cysteine residue (Figure 1A). However, deconjugation via retro-Michael reactions (Figure 1B) and cross-conjugation to endogenous thiol bearing species such as albumin and glutathione have been described for these thiol-maleimide adducts (Baldwin and Kiick, 2011; Jackson et al., 2014; Lahnsteiner et al., 2020). In contrast, hydrolytic succinimide ring opening, also known to occur for maleimide conjugates, results in a more stable structure less likely to undergo retro-Michael reaction (Figure 1B).

Enhanced conjugate stability is generally desired by product developers and significant research efforts have focused on producing more stable maleimide-based bioconjugates (Fontaine et al., 2015; Kalia et al., 2017; Ravasco et al., 2019; Lahnsteiner et al., 2020). Alternatively, mono-sulfone-PEG reagents (Figure 1A) have been suggested to form more stable conjugates. As part of the mono-sulfone PEGylation process, the ketone in the linker is reduced to a secondary alcohol using sodium borohydride post-conjugation (Figure 1C). Prior to reduction the α-keto protons are mildly acidic and can be deprotonated by a base (the first step of a retro-Michael reaction). In the reduced (stabilized) linker these protons are non-acidic and electronically the retro-Michael reaction is a non-viable mechanism of deconjugation. For example, a reduced mono-sulfone-PEG conjugate of a laminin-β peptide was shown to be stable over 12 days at room temperature in a pH 7.5 phosphate buffer; under the same conditions non-reduced mono-sulfone-PEG and maleimide-PEG conjugates exhibited ≈20% and ≈40% deconjugation respectively (Badescu et al., 2014).

Ideally, a HBOC oxygen therapeutic should stay in the vasculature for up to a week. Loss of conjugation would lead to rapid renal clearance of Hb dimers. In this study we therefore compared the efficiency and stability of our previous Hb PEGylation method using maleimide-PEG (Cooper et al., 2020), with a potentially more stable conjugation with a reduced mono-sulfone-PEG.

## Materials and Methods

### Materials

Recombinant protein production in *Escherichia coli* and purification of the Hb mutant termed A12 was as described in detail previously (Cooper et al., 2020). Essentially, BL21 (DE3) cells transformed with pETDuet-1 plasmid containing human Hb with mutant βCys93Ala and αAla19Cys genes were grown in shake flask cultures. Cells were harvested and the Hb protein purified by a combination of cation exchange, anion exchange and affinity chromatography. Samples were stored in liquid nitrogen as stable carbonmonoxy-ferrous Hb (HbCO) adducts and converted to different Hb forms when required (Simons et al., 2018).

20 kDa mono-sulfone-PEG was prepared according to a published synthetic route (Badescu et al., 2014) and 20 kDa maleimide-PEG was purchased from Iris Biotech. A 1 M solution of sodium borohydride, purchased from Sigma-Aldrich, was prepared in 0.1 M NaOH prior to use. All other materials for bioconjugation were supplied by Fisher Scientific, Merck, VWR, GE Healthcare, Sigma-Aldrich and Melford. The PEGylation buffer (100 mM HEPES, 1.2 mM sodium phosphate, 100 mM NaCl, 1 mM EDTA, pH 7.0) was purged with carbon monoxide (BOC) before use. For stability studies reduced glutathione was purchased from Sigma-Aldrich.

### Oxygen Binding

Measurements were carried out essentially as described previously (Cooper et al., 2020) by diluting ferrous oxygenated Hb in 100 mM HEPES, 100 mM NaCl, 1.2 mM sodium phosphate, 1 mM EDTA, pH 7.0. The final protein concentration was 100 μM (heme basis). The oxygen equilibrium curves were measured at 25°C by deoxygenating samples using a helium flow and then equilibrating with different oxygen partial pressures. For each sample, the absorption spectrum was collected to determine the fractional Hb oxygen saturation. Sodium ascorbate and catalase were added to the solution before the titrations to reduce any inactive ferric (met) Hb form and limit its formation during the titration. A titration required about 5 h.

### Mono-Sulfone-PEG Hb PEGylation Process Screening (Figures 2–4)

Hb was maintained in the carboxyhemoglobin form (HbCO) to prevent oxidation during the PEGylation process. Absorption spectra were collected in the range of 450–700 nm and the protein concentration calculated using the molar extinction coefficients for HbCO (Antonini and Brunori, 1971). Three vials of HbCO (0.72 mg, 13.2 μL) were adjusted to 1 mM concentration (on monomer basis) with the CO purged PEGylation buffer (31.8 μL) at pH 7.0. A stock solution of 20 kDa mono-sulfone-PEG (10 mM) in CO PEGylation buffer was prepared immediately prior to use. CO PEGylation buffer and 20 kDa mono-sulfone-PEG in 12:1, 6:1 and 3:1 PEG:Hb tetramer ratios were then added to the protein solutions to give final Hb monomer concentrations of 0.77 mM. PEGylation was then allowed to proceed at 25°C with timepoints taken at 2, 3, 4 and 5.5 h. Each timepoint sample (13 μL) was diluted with 600 mM sodium phosphate, pH 7.5, 150 mM NaCl, 20 mM EDTA buffer (13 μL) and the resulting solutions were cooled in an ice-bath. To the Hb-PEG solutions, 1 M sodium borohydride (1.2 μL) was added, and the reduction mixtures were immersed in ice for 30 min. A second portion of 1 M sodium borohydride (1.2 μL) was added to the reactions and a further 30 min on ice was allowed. Once all timepoint samples had been acquired, analysis was performed by SDS-PAGE using 4–12% Bis-Tris gels using published methods (Cooper et al., 2020).

**FIGURE 2:**
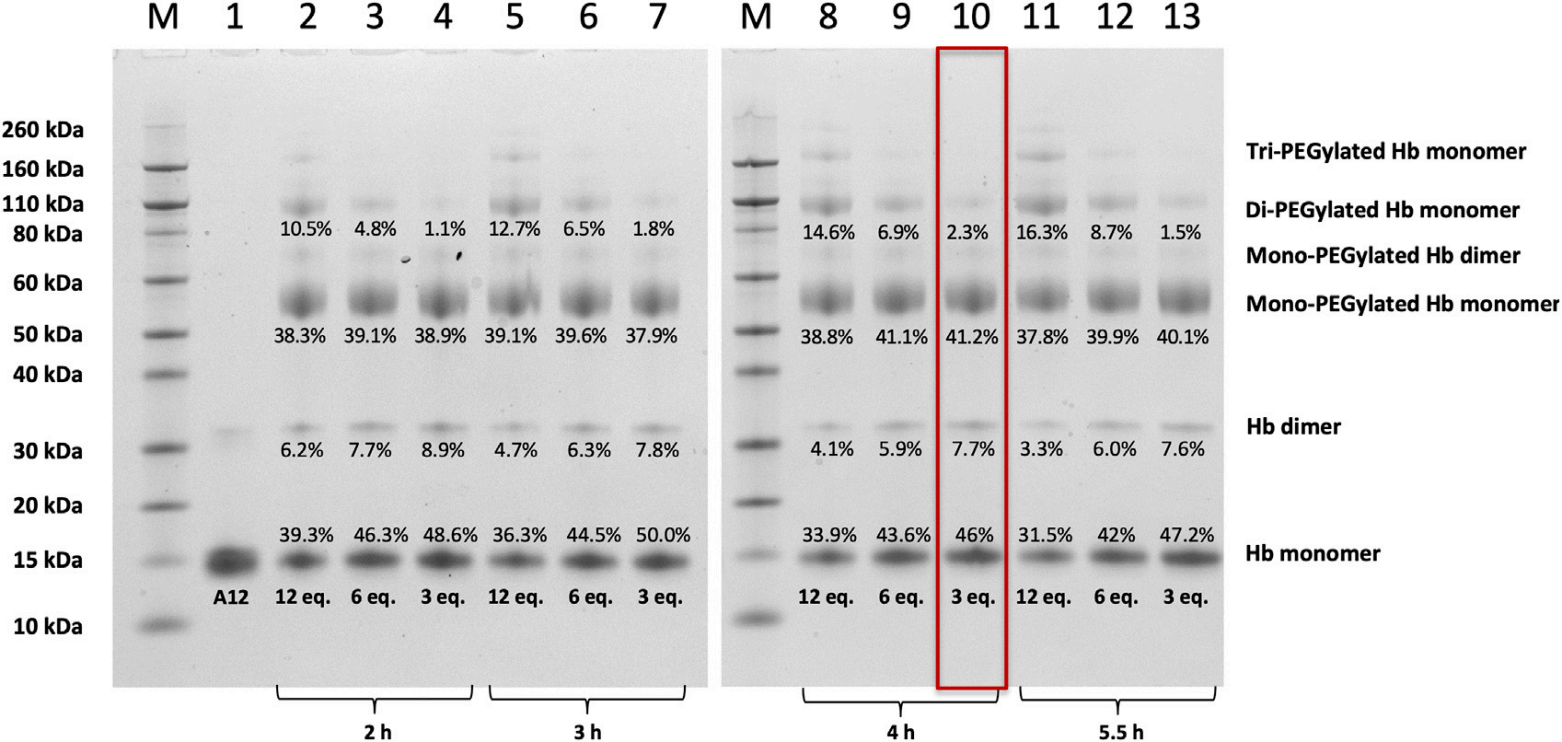
SDS-PAGE of Hb A12 following incubation with mono-sulfone PEG. Gels illustrated before (A12) and after PEGylation with mono-sulfone PEG at different PEG:Hb ratios for different durations of incubation. M = molecular weight markers. Labels equate to % of total protein in lane in each band for: Di-PEGylated Hb monomer; Mono-PEGylated Hb Monomer; Hb dimer; Hb monomer. Enclosed rectangle illustrates conditions chosen for later studies.

**FIGURE 3:**
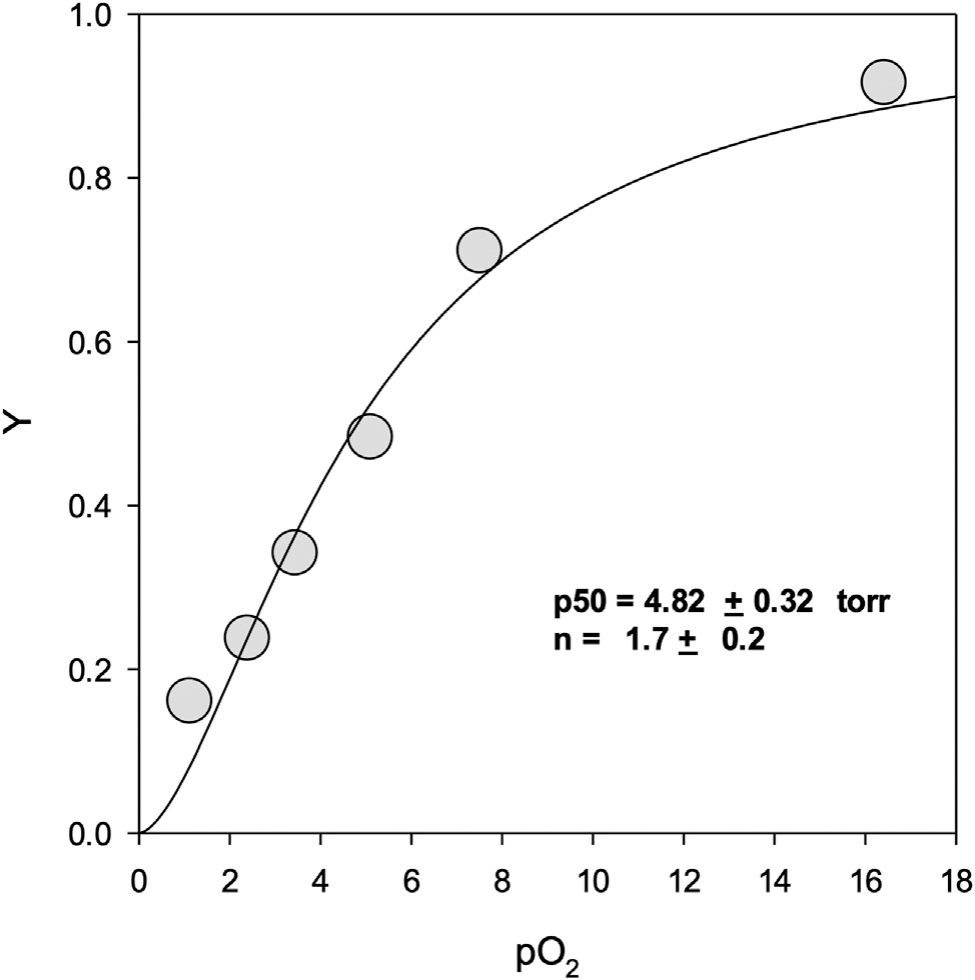
Oxygen equilibrium binding curves. Y represents fractional binding as a function of oxygen partial pressure. Values for binding affinity (p50) and cooperativity (n) are presented ±SEM of the nonlinear regression curve fit to the Hill equation. For experimental conditions see Materials and Methods.

**FIGURE 4:**
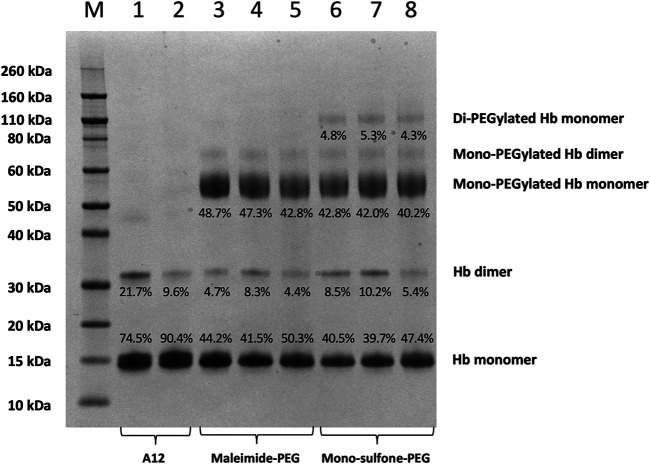
SDS-PAGE comparing A12 conjugation with maleimide-PEG and mono-sulfone PEG. Novex MW markers (M); Hb-A12 (1); Hb-A12 + 10 mM DTT 40°C 1 h incubation (2); maleimide-PEG-A12 reaction mixture (3); maleimide-PEG-A12 product (4); maleimide-PEG-A12 product +10 mM DTT 40°C + 1 h (5); mono-sulfone PEG-A12 reaction mixture (6); mono-sulfone PEG-A12 product (7); mono-sulfone PEG-A12 product +10 mM DTT 40°C + 1 h incubation (8). Stoichiometry for incubation: maleimide-PEG: Hb = 12:1; mono-sulfone-PEG: Hb = 3:1. Labels equate to % of total protein in lane in each band for: Di-PEGylated Hb monomer; Mono-PEGylated Hb Monomer; Hb dimer; Hb monomer.

### PEG-Hb Production for Stability Assessment (Figures 5, 6)

#### Maleimide-PEG Hb

HbCO (5 mg, 91.6 μL) was adjusted to 1 mM concentration (on monomer basis) with CO purged PEGylation buffer (221 μL). A stock solution of 20 kDa maleimide-PEG (10 mM) in CO PEGylation buffer was prepared immediately prior to use. To the protein solution was added 20 kDa maleimide-PEG (20.9 mg, 93.8 μL) in 12:1 PEG:Hb tetramer ratio. PEGylation was then allowed to proceed at 25°C for 2 h. The reaction was quenched by the addition of 3.13 μL of a 0.9 ML-cysteine solution.

**FIGURE 5:**
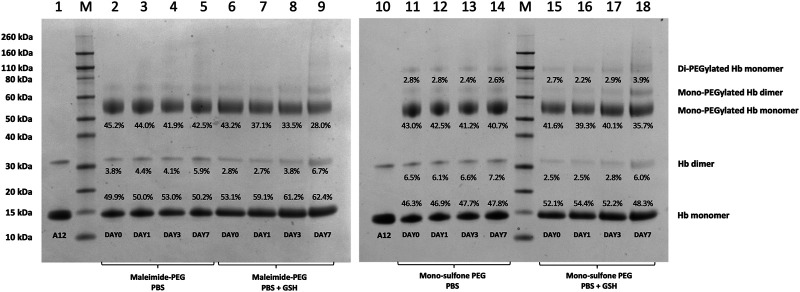
Effect of GSH on stability of Hb-PEG conjugates. SDS-PAGE comparing stability of A12 conjugation with maleimide-PEG and mono-sulfone PEG following incubation for 7 days at 37°C with PBS or 1 mM GSH. Labels equate to % of total protein in lane in each band for: Di-PEGylated Hb monomer; Mono-PEGylated Hb Monomer; Hb dimer; Hb monomer.

**FIGURE 6:**
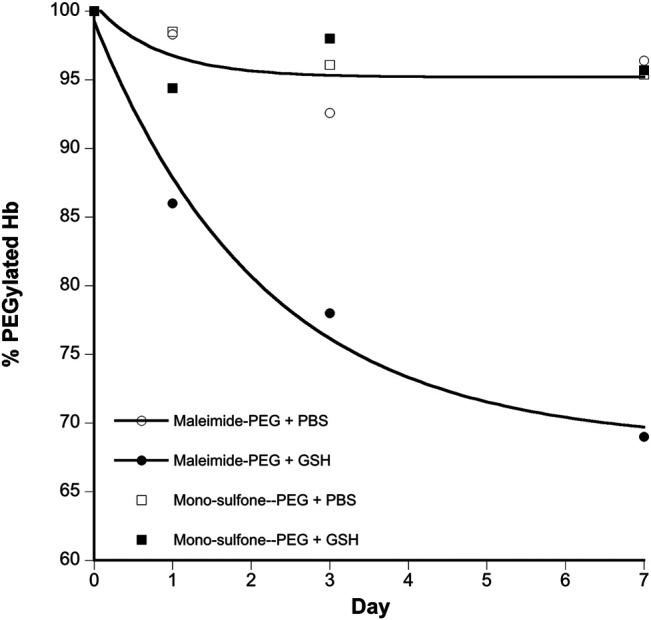
Kinetics of stability of A12 Hb-PEG conjugates. Graphs show the % drop in Hb PEGylation (assuming 100% PEGylation at t = 0). Curve fits illustrated are mono exponential decay for the maleimide-PEG species. The mono-sulfone-PEG curve fits superimpose on the maleimide-PEG + PBS fit so have been removed for clarity. Legend: + PBS maleimide-PEG (o); + PBS mono-sulfone-PEG (□); + 1 mM GSH maleimide-PEG (●), + 1 mM GSH mono-sulfone PEG (■).

#### Mono-Sulfone-PEG Hb

HbCO (5 mg, 91.6 μL) was adjusted to 1 mM concentration (on monomer basis) with CO purged PEGylation buffer (221 μL). A stock solution of 20 kDa mono-sulfone-PEG (10 mM) in CO PEGylation buffer was prepared immediately prior to use. Additional CO PEGylation buffer (70.3 μL) and 20 kDa mono-sulfone-PEG (5.3 mg, 23.4 μL) in 3:1 PEG:Hb tetramer ratio, were added to the protein solution. PEGylation was then allowed to proceed at 25 C for 4 h. The reaction was next diluted with 600 mM sodium phosphate, pH 7.5, 150 mM NaCl, 20 mM EDTA buffer (406 μL) and the resulting solution was cooled in an ice-bath. To the Hb-PEG solution, 1 M sodium borohydride (9.38 μL) was added, and the reduction mixture was immersed in ice for 30 min. A second portion of 1 M sodium borohydride (9.38 μL) was added to the reaction and a further 30 min on ice was allowed.

#### Hb-PEG Purification

Unreacted reagents and unmodified Hb were removed by ultrafiltration/diafiltration (UF/DF) against CO purged PBS using centrifugal filters fitted with 100 kDa MWCO PES membranes. Red permeates were observed due to the presence of unmodified and/or monoPEG Hb. After UF/DF, absorption spectra were collected to check concentration and oxidation state. The samples were then frozen and stored at −80°C.

#### Hb-PEG Stability in PBS Containing 1 mM Reduced Glutathione (GSH)

Hb-PEG solutions were prepared at 0.5 mg/mL in CO purged PBS buffers containing no additive or 1 mM reduced GSH. Each sample was supplemented with 0.05% w/v sodium azide. Aliquots (60 μL) of the samples were then prepared. One aliquot per sample was directly frozen at −80°C (t = 0 days). The remaining aliquots were incubated at 37°C. At t = 1, 3 and 7 days samples were removed from storage at 37°C and frozen at −80 °C. Once all timepoint samples had been acquired, the samples were thawed and analyzed by SDS-PAGE using 4–12% Bis-Tris gels as previously described (Cooper et al., 2020).

#### Data Processing Methodology

The stabilities of maleimide- and mono-sulfone PEG Hb conjugate linkages were assessed by evaluating the levels of the Hb-PEG and free Hb bands observed by SDS-PAGE gel band densities in isolation (i.e., ignoring all other species present). For normalization and to aid comparison, the percentage of Hb-PEG was expressed as a relative percentage compared to the t = 0 days value in each analysis.

## Results

In the recombinant human Hb mutant studied (A12), the βCys93Ala mutation removes the only native reactive cysteine in Hb on the β subunit near the α/β interface and the αAla19Cys mutation creates a new reactive cysteine on the α subunit. This new cysteine is at a surface site distant from the dimer/dimer interface and heme pocket, and hence less likely to affect protein function than βCys93.

Figure 2 shows SDS-PAGE gels of unmodified A12 and A12 following treatments with different amounts of a mono-sulfone PEG for different durations. SDS separates the 4 non-covalently bound subunits of A12 (α2β2) into 16 kDa α and β monomers. A small fraction of A12 runs as a covalently bonded dimer, presumably either due to oxidative deamination and intramolecular cross-linking during expression in *E. coli* (Levine et al., 1998) or a new disulfide bridge between the αAla19Cys residues in the two dimers (Cooper et al., 2020) Following PEGylation with 20 kDa mono-sulfone PEG, a significant new band is observed at 56 kDa and smaller bands at ca. 70 and 100 kDa, and - at high PEG:Hb ratios - bands >150 kDa. Given that PEGylated Hb adducts run at higher apparent molecular weight than predicted from unPEGylated protein markers (Caccia et al., 2009), the 56 kDa band is consistent with mono-PEGylation of A12 at Ala19Cys as was previously seen following maleimide-PEGylation (Cooper et al., 2020). The band at 70 kDa is likely mono-PEGylated Hb dimer and the 100 kDa band is consistent with di-PEGylated Hb monomer, with non-specific PEGylation at a second site evident. The bands >150 kDa presumably represent additional non-specific PEGylation sites only populated at PEG:Hb ratios >6:1.

As the denaturing gel separates the Hb α_2_β_2_ tetramer into α and β monomers, Hb with 100% mono-sulfone PEG attachment to the two αAla19Cys subunits will run as two bands of equal density: the 16 kDa band of the two unPEGylated β monomers and the 56 kDa band of the two PEGylated α monomers. Consistent with this an approximate 50:50 ratio between the protein intensity at the 16 kDa and 56 kDa bands is indeed seen at PEG:Hb ratios from 3–12 and incubation durations from 2–5.5 h. The longer the incubation time and the higher PEG:Hb ratio, the closer the ratio comes to 50:50. However, this is at the expense of a significant increase in the fraction of Hb that is PEGylated at multiple sites. Therefore for future studies a compromise was chosen of an incubation at 3:1 PEG:Hb ratio for 4 h, leading as it does to >80% yield of the desired mono PEGylated product with less than 2.5% di-PEGylation.

Figure 3 shows the oxygen binding curve for mono-sulfone PEG A12. Under “pseudophysiological” conditions (see Methods), the oxygen affinity was 4.82 ± 0.32 Torr with a Hill coefficient for cooperative oxygen binding of 1.7 ± 0.2. This is broadly in line with recombinant Hb for this mutant measured under the same conditions, with or without maleimide-PEGylation (Cooper et al., 2020). This indicates that, like maleimide-PEG, the mono-sulfone-PEG conjugate of A12 has no dramatic effect on the functional properties of the protein.

Figure 4 directly compares the efficiency of A12 PEGylation with maleimide-PEG or mono-sulfone-PEG. Both PEG reagents behave similarly although the mono-sulfone-PEG does show a significant fraction of di-PEGylated product. A brief incubation with competing thiol (dithiothreitol, DTT) at 40°C reduced some of the covalent dimers in the A12 starting material, most likely by breaking interdimer disulfide bridges. However, this incubation at 40°C also seemed to partially degrade the maleimide-PEG product. Therefore a longer term stability study at physiological temperatures was performed in the presence and absence of GSH (Figure 5). Both maleimide-PEG and mono-sulfone-PEG conjugates showed good stability following incubation in phosphate buffered saline (PBS) for seven days at 37°C, with very little (< 5%) decrease in the mono-PEGylated product. However, in the presence of 1 mM GSH, PEG deconjugation was faster. Figure 6 shows a side-by-side comparison of the stability of A12 Hb-maleimide-PEG and A12 Hb-mono-sulfone-PEG over the seven-day period. In the mono-sulfone-PEG A12 the loss was < 10% even in the presence of a competing thiol. In contrast, in maleimide-PEG A12 there was greater deconjugation, with a >30% loss in PEGylation after seven days in the presence of GSH.

## Discussion

### Specificity of Conjugation Reaction

This paper demonstrates that it is possible to engineer a new sulfhydryl residue on the surface of a clinically relevant protein (Hb) that can be conjugated efficiently by two different methods. The genetically engineered cysteine residue that replaced Alanine19 on the Hb α subunit, resulted in >85% mono PEG product using either maleimide-PEG or mono-sulfone PEG (Figure 5). However, there were some differences between the two methods. The mono-sulfone Hb had considerable cross-reactivity at other sites if used at the same concentration ratio as maleimide-PEG (12:1 PEG:Hb ratio). In the worst-case scenario (12:1 PEG:Hb ratio for 5.5 h) almost 1/3 of the PEGylated protein was conjugated at more than one site per Hb dimer, resulting in a significant fraction of di (and higher) PEGylated products (Figure 2). This reactivity must be at a different Hb residues to the cysteine engineered at the α19 residue.

Of the three cysteine residues in native human Hb, the surface accessible βCys93 is readily reactive to sulfhydryl reagents, such as iodoacetamide (Guidotti and Konigsberg, 1964) or p-hydroxymercuribenzoic acid (PMB) (Rosemeyer and Huehns, 1967). As the concentration of PMB is increased, further modification can occur, first at αCys104 and then at βCys112 (Kan et al., 2013). Our A12 recombinant Hb contains the βCys93Ala mutation so PEGylation at βCys93 cannot occur and PEGylation is at first favoured at the newly introduced surface site αAla19Cys (Cooper et al., 2020). It is possible that higher concentrations of mono-sulfone-PEG are increasingly able to modify the more inaccessible αCys104 and the βCys112 residues, resulting in higher order PEG species.

As Hb runs as a monomer on a denaturing gel - and A12 has two cysteine on its α subunit and one on its β subunit - the highest order PEGylation observed on a denaturing gel for the reaction of mono-sulfone-PEG solely at A12 cysteines would be di-PEGylation (at αCys19 and αCys104). Yet at high PEG:Hb ratios, a tri-PEGylated species is clearly observed (Figure 2). This requires PEGylation at a different site than cysteine. Given the almost complete absence of higher order species following conjugation with maleimide-PEG (Figure 4), this site is likely to be one that is both surface accessible and available for preferable conjugation by mono-sulfone PEG over maleimide-PEG. At neutral pH, maleimide-PEG is far more reactive at sulfhydryl than other nucleophilic residues such as amines. However, this is less true for mono-sulfone PEG; indeed bis-sulfone-PEGs similar to those described in this paper have been shown to react with polyhistidine tags in proteins (Cong et al., 2012). Of the potential surface primary amines or histidine on Hb, of note are the significant number of reactive lysine residues. These were taken advantage of in the PEGylation method developed by Sangart Inc. for their oxygen therapeutic Hemospan (MP4, MalPEG-Hb), which involved reaction at surface lysine residues by 2-iminothiolane, followed by conjugation of the resulting thiols using maleimide-PEG (Vandegriff et al., 2008). Ten surface lysine residues were found to be reactive to 2-iminothiolane; three on the α subunit (αLys7, αLys16, and αLys40) and seven on the β subunit (βLys8, βLys17, βLys59, βLys66, βLys95, and βLys132). It is therefore reasonable to assume that at least some of the higher order di- and tri- PEGylated species observed involve conjugation of mono-sulfone-PEG to one of these surface lysines.

One option to decrease the formation of these higher order conjugates would be to remove the reactive cysteine and/or amine residues by site directed mutagenesis, always assuming of course that protein function is conserved. In terms of cysteines, βCys112 is situated near the α/β interface. However, it is not highly conserved in mammalian Hbs and replacing βCys112 with glycine has minimal effects on protein structure and function (Vasquez et al., 1999). Although the β112 Asp, Ser, Thr or Val mutations do result in proteins with differences in the dimer/tetramer equilibrium, they still retain largely unchanged oxygen transport properties (Yamaguchi et al., 1998). Databases of human Hb polymorphisms reveal some protein instability in Hb from people with βCys112 mutations (Giardine et al., 2020). However, whilst βCys112Arg (Hb Indianapolis) is linked to thalassemia (Adams et al., 1979), there are few, or no, clinical symptoms for people with βCys112Gly (Hb Saint-Marcelin), βCys112Phe [Hb Canterbury, (Brennan et al., 2002)], βCys112Tyr [Hb Yahata, (Harano et al., 1991)] and βCys112Trp [Hb Toranomon, (Harano et al., 1996)]. Therefore, it seems entirely feasible to substitute the βCys112 residue using recombinant techniques, if it were deemed important to decrease further non-specific PEGylation reactions in a clinical product. An alternative to removing βCys112 in adult Hb is to use the strongly homologous fetal Hb (HbF), as starting material (Ratanasopa et al., 2015; Simons et al., 2018). HbF is an α_2_γ_2_ tetramer; the γ subunit of HbF has no cysteines other than γCys93, making the mutant γCys93Ala completely unreactive to sulfhydryl reagents.

In contrast to βCys112Phe, αCys104 is highly conserved across many Hb species and might be more difficult to replace. Clinical data support this (Giardine et al., 2020). The Hb chain in humans is coded for in two almost identical genes (HbA1 and HbA2). Hemoglobin polymorphisms reveal severe protein instability in Hb from people with Cys104 mutations in their HbA1 or HbA2 genes. Reported mutations include HbA2 αCys104Arg [Hb Iberia, (Bento et al., 2012)], HbA2 αCys104Tyr [Hb Sallanches, (Morle et al., 1995)], HbA1 αCys104Ser [Hb Oegstgeest, (Harteveld et al., 2005)] and HbA1 αCys104Trp (alpha codon 10 TGC-TGG). In these cases, the protein is too unstable to be detectable in heterozygotes or associated with thalassemia in homozygotes.

In general αCys104 is the most inaccessible of the three cysteines in adult Hb to chemical modification with the least solvent accessible surface area (Mitra et al., 2017). Even when it is somewhat more accessible than βCys112 to modification [e.g. by PMB (Kan et al., 2013)], this is likely due to structural changes in Hb produced by PMB first reacting at βCys93 facilitating dissociation of the α/β dimer into monomers (Rosemeyer and Huehns, 1967). As a result, αCys104 is likely to remain inaccessible to PEGylation in Hb mutants such as A12, which lack βCys93. Therefore even if it were possible to produce a stable mutated protein, it would likely not be advantageous to mutate this residue to decrease non-specific PEGylation reactions.

Any side reactions due to conjugation by mono-sulfone-PEG at surface amines would be harder to alter by site directed mutagenesis, given the larger number of potentially reactive sites (including at least ten lysines as previously noted). A first step would be to address the scale of the problem by identifying the specific lysines modifiable by mono-sulfone-PEG (Iafelice et al., 2007; Vandegriff et al., 2008) and then determining if they were essential to protein function. However, a more practical solution is likely to be to optimize the incubation conditions. Lowering the PEG:Hb ratio and shortening the incubation time decreased the ratio of diPEGylated Hb to monoPEGylated Hb to approximately 5% (Figure 2). Fine tuning of the PEGylation condition could decrease these side reactions even further (Badescu et al., 2014). One option is to lower of the pH in the incubation medium below the current 7.0; this could increase the specificity of the sulfhydral reactivity of the mono-sulfone PEG by suppressing reactivity with amino groups.

### Stability of Conjugation Product

The two PEGylation methods differed significantly in terms of stability of the mono-PEGylated form in the presence of a competing thiol (Figure 6). The mono-sulfone PEG was resistant to deconjugation for seven days at 37°C, retaining over 95% conjugation in the presence of both PBS and 1 mM GSH. In contrast the maleimide-PEG form of A12 retained only about 70% conjugation after incubation with 1 mM GSH. In all cases the maleimide-PEG deconjugation approached a minimum after seven days with a significant fraction of protein conjugate still intact; it is possible that that during the incubation period, a sub-portion of the maleimide-PEG conjugate undergoes hydrolytic ring opening, contributing to the stability observed.

In this study we have clearly shown that mono-sulfone PEG provides an efficient conjugate that is more stable than maleimide-PEG. Alternative conjugate stabilization methods are available. The maleimide-PEG bond itself can be stabilised if the succinimide moiety of a maleimide−thiol conjugate is hydrolyzed, as the ring-opened product is resistant toward cleavage (Fontaine et al., 2015); thiomaleimides can be stabilized by light-triggered ring hydrolysis (Kalia et al., 2017) and the thiosuccinimide by transcyclization reactions (Lahnsteiner et al., 2020). As well as mono-sulfone PEG, alternatives to maleimide-PEG as the conjugate starting material include exocyclic maleimides (Kalia et al., 2016), and carbonylacrylic reagents (Bernardim et al., 2016). Also 2-formylphenylboronic acids (Li et al., 2020) provide stable alternatives compared to maleimide-PEG.

The relative stability results in our paper are in broad agreement with a previous study comparing mono-sulfone-PEG and maleimide-PEG conjugation to a single cysteine residue on Lamβ_925−933_, a synthetic linear nona-peptide corresponding to residues 925–933 of the laminin β1 chain (Badescu et al., 2014). In that study, there was no deconjugation of the mono-sulfone-PEG adduct over a twelve-day incubation period. However, there was 40% deconjugation of the maleimide-PEG adduct, with most of that occurring in the first three days. The work reported in the current paper is more significant, however. The Lamβ_925−933_ stability study was at room temperature in pH 7.5 phosphate buffer. However, Figure 6 shows that a mono-sulfone PEG adduct to a full length physiologically relevant protein (Hb) shows minimal deconjugation at a physiological ionic strength (PBS) and physiological temperature (37 °C), even in the presence of competing thiols at higher than physiological (1 mM GSH) concentrations.

### Physiological Relevance of Enhanced Stability of the Conjugate

How relevant is the enhanced stability these alternative PEGylation methods provide compared to the more common maleimide-PEG method? For drug conjugates requiring relatively short *in vivo* lifetimes, the lifetime of the thioether conjugate bond formed using maleimide-PEG frequently exceeds the desired *in vivo* lifetime of the drug conjugate. However, when the desired lifetime of the drug is weeks to months, as in some antibody drug conjugates, thiol exchange rates can exceed their intended *in vivo* lifetime resulting in the premature loss of active drug and/or the formation of a toxic unconjugated drug (Shen et al., 2012).

What is the lifetime for Hb when formulated as an oxygen therapeutic? In normal physiological situations, Hb is packed inside red blood cells and shares the lifetime of those cells, with a mean of 115 days (Franco, 2012). However, outside the red blood cell this lifetime is considerably shorter; when infused, cell free Hb has a limited lifetime of ∼0.5–1.5 h (Savitsky et al., 1978). This is due to the dissociation of Hb into dimers. These can readily pass through the kidney glomerular filtration barrier causing hemoglobinuria (Savitsky et al., 1978), or be scavenged by the monocyte/macrophage CD163 receptor - either directly or following binding to the acute phase protein haptoglobin - enabling subsequent removal from the circulation by the liver or spleen (Schaer et al., 2006). A major goal of any HBOC is therefore to increase the size of Hb and increase vascular retention. This goal has been achieved in covalently cross-linked Hb, polymerized Hb, vesicular encapsulated Hb and PEGylated Hb (Bettati and Mozzarelli, 2011). However, in the case of historical and current HBOCs the *in vivo* lifetime in patients and animal models appears unconnected to the method of HBOC production. For example studies in patients indicate half-lives of 3–24 h for cross-linked Hb, 8.5–22 h for polymerized Hb and 14–66 h for PEGylated Hb (Taguchi et al., 2017). Hb vesicles have not been infused in humans, although the prediction from animal models is that the half-life would be of the same order of magnitude, i.e. no more than three days (Sou et al., 2005).

The similarity of vascular half-life independent of HBOC production method suggests that the strategy of increasing the size of the Hb dimer to prevent renal filtration is largely irrelevant to *in vivo* lifetime; HBOCs are cleared via the spleen and liver and not just the kidney (Keipert et al., 1994). This suggests that PEGylated HBOCs are cleared primarily by phagocytosis rather than by filtration in the kidney following loss of conjugation. It therefore seems unnecessary to develop an alternative to maleimide-PEG for the current use of HBOC as an oxygen therapeutic targeted for acute use.

However, red blood cells are not just transfused in acute situations. Although in high-income countries, transfusion is commonly used for cardiovascular surgery, transplant surgery and trauma, a significant fraction (>50%) is used in tumor therapy and for the treatment of blood diseases (Tinegate et al., 2016). Also, although rarely used to treat anemia in higher income countries, this is one of the most common uses of transfusions in lower and middle income countries, especially in children (Maitland et al., 2019). The use of HBOC in these non-acute situations would require a significantly longer lasting HBOC, closer to the vascular lifetime of a red blood cell. The creation of a more stable PEG conjugation might then become relevant as part of a strategy to create a long lasting red blood cell substitute. In that scenario, the enhanced stability of the mono-sulfone PEG linkage demonstrated in this paper could become an important part of the solution.

## Data Availability

The original contributions presented in the study are included in the article/supplementary files, further inquiries can be directed to the corresponding author.

## Abbreviations

DTT, dithiothreitol; GSH, reduced glutathione; Hb, Hemoglobin; HBOC, Hemoglobin Based Oxygen Carrier; A12, recombinant adult human Hb with βCys93Ala and αAla19Cys mutations; HbCO, carbon monoxide bound Hb (carboxyhemoglobin); PBS, phosphate buffered saline; PEG, poly(ethylene glycol); PMB, p-hydroxymercuribenzoic; SDS-PAGE, sodium dodecyl sulfate polyacrylamide gel electrophoresis.
